# Inter-practice variation in diagnosing hypertension and diabetes mellitus: a cross-sectional study in general practice

**DOI:** 10.1186/1471-2296-10-6

**Published:** 2009-01-21

**Authors:** Markus MJ Nielen, François G Schellevis, Robert A Verheij

**Affiliations:** 1 NIVEL (Netherlands Institute for Health Services Research), P.O. Box 1568, 3500BN Utrecht, the Netherlands; 2 Department of General Practice/EMGO Institute, VU University Medical Center, Amsterdam, the Netherlands

## Abstract

**Background:**

Previous studies of inter-practice variation of the prevalence of hypertension and diabetes mellitus showed wide variations between practices. However, in these studies inter-practice variation was calculated without controlling for clustering of patients within practices and without adjusting for patient and practice characteristics. Therefore, in the present study inter-practice variation of diagnosed hypertension and diabetes mellitus prevalence rates was calculated by 1) using a multi-level design and 2) adjusting for patient and practice characteristics.

**Methods:**

Data were used from the Netherlands Information Network of General Practice (LINH) in 2004. Of all 168.045 registered patients, the presence of hypertension, diabetes mellitus and all available ICPC coded symptoms and diseases related to hypertension and diabetes, were determined. Also, the characteristics of practices were used in the analyses. Multilevel logistic regression analyses were performed.

**Results:**

The 95% prevalence range for the practices for the prevalence of diagnosed hypertension and diabetes mellitus was 66.3 to 181.7 per 1000 patients and 22.2 to 65.8 per 1000 patients, respectively, after adjustment for patient and practice characteristics. The presence of hypertension and diabetes was best predicted by patient characteristics. The most important predictors of hypertension were obesity (OR = 3.5), presence of a lipid disorder (OR = 3.0), and diabetes mellitus (OR = 2.6), whereas the presence of diabetes mellitus was particularly predicted by retinopathy (OR = 8.5), lipid disorders (OR = 2.8) and hypertension (OR = 2.7).

**Conclusion:**

Although not the optimal case-mix could be used in this study, we conclude that even after adjustment for patient (demographic variables and risk factors for hypertension and diabetes mellitus) and practice characteristics (practice size and presence of a practice nurse), there is a wide difference between general practices in the prevalence rates of diagnosed hypertension and diabetes mellitus.

## Background

The prevalence rate of chronic diseases like hypertension and diabetes mellitus is rapidly increasing, particularly in industrialized countries [1,2]. Since hypertension and diabetes mellitus are major risk factors for the development of cardiovascular and renal diseases, and could result in premature death [3,4], detection and treatment of these diseases is warranted. In the Netherlands, most hypertensive and diabetic patients are detected and treated by a General Practitioner (GP). Dutch GPs have a gatekeeper role for access to specialized care. All Dutch inhabitants are listed with a general practice and generally the GP is the first professional to be consulted for health problems.

According to the guidelines of the Dutch College of General Practitioners, measurement of blood pressure and blood glucose are recommended in specific patient groups [5,6]. It could be hypothesized that when all GPs are following these guidelines, there should be no variance between general practices, i.e. inter-practice variation, in the prevalence rates of diagnosed hypertension and diabetes mellitus after adjustment for patient characteristics in a practice. Demographic characteristics of the patients in a general practice, like age and gender, can influence the prevalence rates of diagnosed hypertension and diabetes mellitus in a practice, but the prevalence rates can also be affected by an unequal distribution of risk factors between populations of different practices.

Previous studies of inter-practice variation of the prevalence rate of hypertension and diabetes mellitus showed a wide variation at practice level. However, in these studies inter-practice variation was calculated without controlling for clustering of patients within practices and without adjusting for patient and practice characteristics [7-11]. Multilevel analyses takes the statistical dependency of patients within practices into account. Furthermore, inter-practice variance can be adjusted for factors influencing prevalence rates at the level of patients and practices. Usually this results in fewer significant findings at the higher hierarchical level (in this case: practices).

Therefore, the purpose of this study is, first, to assess inter-practice variation of diagnosed hypertension and diabetes mellitus prevalence rates using a multi-level design. Second, we will study to what extent practice variation can be explained by specific characteristics of the practices involved at either the patient level (such as age, gender and the presence of risk factors), or in terms of practice characteristics (such as practice type or the level of practice support personnel).

## Methods

### Study population

Data were used from the Netherlands Information Network of General Practice (LINH). These data were retrieved from electronic medical records kept by a representative sample of 75 GP practices in 2004. Data include information on consultations, morbidity, prescriptions and referrals. Practices as well as patients are representative for the Dutch population [12,13]. Patients under 25 were excluded, because of their low probability of having hypertension and/or diabetes mellitus. Practices who recorded during less than six months in 2004 were excluded from statistical analyses.

The study was carried out according to Dutch legislation on privacy. The privacy regulation of the study was approved by the Dutch Data Protection Authority. According to Dutch legislation, nor obtaining informed consent nor approval by a medical ethics committee was obligatory for observational studies.

### Classification of hypertension and diabetes mellitus

Morbidity data were derived from consultation diagnoses and furthermore from all prescriptions issued by the participating practices. Diagnoses were recorded using the ICPC-1 coding system (International Classification of Primary Care) [14]. When issuing a prescription, a diagnostic code was recorded, and the selected drug was automatically linked to the Anatomical Therapeutic Chemical (ATC) Classification System. [15].

Patients were classified as hypertensive when one of the following criteria was fulfilled: 1) diagnosed at any consultation as uncomplicated hypertension or as hypertension with involvement of target organs (ICPC code K86 and K87, respectively) and/or 2) any prescription of one of the following medicines for hypertension: antihypertensives (ATC code C02), diuretics (ATC code C03), beta-blockers (ATC code C07), calcium blockers (ATC code C08) and/or ACE-inhibitors (ATC code C09). Patients were classified as diabetic when the GP recorded the diagnosis diabetes mellitus (ICPC code T90) at any consultation or prescribed insulin (ATC code A10A) or oral blood glucose lowering drugs (ATC code A10B). It was not possible to discriminate between diabetes mellitus type 1 and type 2, because there are no different ICPC codes for these types.

The prevalence rates of diagnosed hypertension and diabetes mellitus were calculated by counting the number of patients who fulfilled the above mentioned criteria in 2004 or in any previous year (up to 1997 when LINH started). The total number of patients registered in the participating general practices in 2004 was used as the denominator. The prevalence rates of diagnosed hypertension and diabetes mellitus were calculated for men and women separately in the following age groups: 25–44 years, 45–64 years, 65–74 years and 75 years and older.

### Patient and practice characteristics

Patient and practice characteristics were used to study which factors could explain the inter-practice variation of diagnosed hypertension and diabetes mellitus prevalence rates. The patient characteristics consisted of demographic and morbidity data. Demographic characteristics of the patients in a general practice can influence the prevalence rates of diagnosed hypertension and diabetes mellitus in a practice, but the prevalence rates can also be affected by an unequal distribution of risk factors between populations of different practices. The demographic variables were age and gender. Furthermore, type of health care insurance (public or private) was included as an indicator for socio-economic status, derived from the electronic medical records (the 60% people with lower incomes in the Netherlands are publicly insured, the 40% people with higher incomes are privately insured).

We also included a number of risk factors for hypertension and diabetes mellitus at patient level. The morbidity variables were all symptoms and diseases in case of which the GP should measure blood pressure and/or blood glucose according to the guidelines of the Dutch College of General Practitioners. According to these guidelines, blood pressure should be measured in 1) patients with symptoms related to a cardiovascular disease, 2) patients with cardiovascular disease, 3) patients with an increased risk of developing a heart disease and 4) patients who use a drug with cardiovascular side effects [5]. Blood glucose should be measured in 1) patients with symptoms related to diabetes mellitus, 2) diseases caused by diabetes mellitus and 3) patients aged 45 years and older with risk factors for diabetes mellitus [6]. The used ICPC coded symptoms and diseases are presented in table 1.

**Table 1:** ICPC coded symptoms and diseases

**Hypertension**	**Diabetes mellitus**
Symptoms of cardiovascular disease (K01-K29)^1^	
Ischemic heart disease with angina (K74)	Ischemic heart disease with angina (K74)
Heart failure (K77)	Heart failure (K77)
Stroke/cerebro-vasculair accident (K90)	Stroke/cerebro-vasculair accident (K90)
Atherosclerosis/PVD (K92)	Atherosclerosis/PVD (K92)
	Hypertension^2^
	Excessive thirst (T01)
Obesity (T82)	Obesity (T82)
Overweight (T83)	Overweight (T83)
Lipid disorder (T93)	Lipid disorder (T93)
	Retinopathy (F83)
Renal disease (symptoms or chronic disease)^3^	Renal disease (symptoms or chronic disease)^3^
Diabetes mellitus^2^	

^1 ^At least one of the following symptoms: Heart pain (K01), Pressure/Tightness of heart (K02), Cardiovascular pain not otherwise specified (K03), Palpitations/awareness of heart (K04), Irregular heartbeat other (K05), Prominent veins (K06), Swollen ankles/oedema (K07), Fear of heart disease (K24), Fear of hypertension (K25), Fear of cardiovascular disease other (K27), Limited function/disability (K28), Cardiovascular symptoms/complaints other (K29).

^2 ^As described in section 'Classification of hypertension and diabetes mellitus'

^3 ^At least one of the following symptoms/diseases: Pyelonephritis/pyelitis (U70), Malignant neoplasm of kidney (U75), Malignant neoplasm of bladder (U76), Malignant neoplasm urinary other (U77), Neoplasm urinary tract not otherwise specified (U79), Injury urinary tract (U80), Congenital anomaly urinary tract (U85), Glomerulonephritis/nephrosis (U88), Urinary calculus (U90), Abnormal urine test not otherwise specified (U98), Urinary disease other (U99)

Differences in the size and the organization structure of practices can also influence the prevalence rates of diagnosed hypertension and diabetes mellitus in the practices. Therefore the following practice characteristics were used in the analyses: type of practice (solo, duo, group or health centre) and the presence of a practice nurse. It was not possible to use variables at GP level, since GPs in group practices and health centers usually do not have personal patient lists.

### Statistical analyses

Multilevel logistic regression analyses with a random intercept were performed using the second order PQL method [16]. The aim of our study is the inter-practice variation, which is estimated by the between practice variation in the multilevel models. This variation is due to differences in the practice population (for instance differences in age or gender structure of the practice population) and to contextual effects, such as environmental effects (for instance degree of urbanization or local culture) or practice effects (for instance attitudes of doctors or staffing of the practice). For both dependent variables (the presence of diagnosed hypertension and diabetes mellitus), three logistic regression models were calculated: 1) a null model with only the intercept, 2) model 1 plus patient characteristics 3) model 2 plus practice characteristics. For all models the between practice variation and the intercept of the model were used to estimate the 95% prevalence range for the practices (intercept plus and minus 1.96 times the square root of the between practice variation and transformed back from a logit scale). Covariates were added (grand mean centered) to the model to explain the between practice variation. The range after the most complex model (model 3), is an indication of the left over, unexplained, inter-practice variation that could not be explained by the covariates. Age was added to the model as a polynomial function, since the association of age with the dependent variables was non-linear.

Model 3 was also used to determine which patient and general practice characteristics were the best predictors of the existence of diagnosed hypertension and diabetes mellitus in an individual patient by calculating odds ratio's (ORs). All statistical analyses were performed with MLwiN, a statistical program for multilevel analyses [17].

## Results

### Population characteristics

In 2004, data of 58 out of the 75 (77%) participating LINH practices could be used for statistical analyses, including 168.045 registered patients. 17 practices had to be excluded because of (partly) missing data. The characteristics of the studied population were representative for the Dutch population in terms of gender and age (Statistics Netherlands, ; data not shown).

### Prevalence of hypertension and diabetes mellitus

The prevalence rates of diagnosed hypertension and diabetes mellitus in 2004, classified by age group and gender, are shown in table 2. The mean prevalence rate of diagnosed hypertension was 142.0 per 1000 persons. In all age categories, the prevalence rate of diagnosed hypertension was higher for women compared with men. The highest prevalence rate of diagnosed hypertension was found in 75+ aged women (448.6/1000 persons). The mean prevalence rate of diagnosed diabetes was 60.3 per 1000 persons in 2004. The prevalence rate of diagnosed diabetes mellitus was higher in women compared to men, except for the age category 45–64 years. The highest prevalence rate was found for 75+ aged women (190.7/1000 persons).

**Table 2:** The prevalence of hypertension and diabetes mellitus in general practice, classified by age and gender in 2004 (per 1000 persons).

	**Total (% CI)**		**25–44 years**		**45–64 years**		**65–74 years**		**75+ years**	
			
			**M**	**F**	**M**	**F**	**M**	**F**	**M**	**F**
**Hypertension**	142.0	(140.3 – 143.6)	22.8	29.7	152.2	174.1	306.6	376.9	341.4	448.6
**Diabetes Mellitus**	60.3	(59.2 – 61.5)	12.0	12.2	69.1	57.4	151.6	159.3	165.0	190.7

### Inter-practice variation of hypertension and diabetes mellitus

Multilevel logistic regression models for the prevalence rates of diagnosed hypertension and diabetes mellitus are shown in table 3 and 4 (see Additional file 1). The intercept of the model and the variance at practice level were used to calculate the 95% prevalence range for the practices in the three models. In model 1 the prevalence rate of diagnosed hypertension and diabetes mellitus in practices varied between 77.9 and 238.7 per 1000 patients and between 30.0 and 109.7 per 1000 patients, respectively. For hypertension, the variance at practice level dropped 24.1% after adjustment for patient and practice characteristics, resulting in a range between practices of 66.3 to 181.7 per 1000 patients. The variance at practice level decreased 32.3% for diabetes mellitus after adding patient and practice characteristics to the model, resulting in a inter-practice range of 22.2 to 65.8 per 1000 patients.

The most important predictors of having a diagnosis of hypertension were the following patient characteristics: obesity (OR = 3.5), presence of a lipid disorder (OR = 3.0), and the presence of diabetes mellitus (OR = 2.6). There were no statistically significant associations between practice characteristics and the presence of hypertension.

Retinopathy (OR = 8.5), lipid disorders (OR = 2.8) and hypertension (OR = 2.7) were the most important predictors of the presence of diabetes mellitus. Of all practice characteristics, only practice type was associated with diabetes mellitus (OR = 1.3). In a group practice the chance of having diabetes mellitus was higher compared with solo practices.

## Discussion

The inter-practice variation of the prevalence rates of diagnosed hypertension and diabetes mellitus was 66.3 to 181.7 per 1000 patients and 22.2 to 65.8 per 1000 patients, respectively, after adjustment for patient and practice characteristics. The presence of diagnosed hypertension and diabetes was best predicted by patient characteristics. The most important predictors of hypertension were the presence of obesity, a lipid disorder and diabetes mellitus, whereas the presence of diabetes mellitus was particularly predicted by retinopathy, lipid disorders and hypertension.

Inter-practice variation for the prevalence rates of hypertension and diabetes mellitus has been studied previously, but without using multi-level statistical techniques [7-11]. We expected that the variation between practices would be lower if the appropriate multilevel techniques were applied. However, the results of the present study are fully in line with these previous studies and still show a wide inter-practice variance of the prevalence of diagnosed hypertension and diabetes mellitus, even after correcting for the characteristics of the practice populations.

Ideally, there should be no practice variation in the prevalence rates of diagnosed hypertension and diabetes mellitus after adjustment for demographic, socio-economic or case-mix variables. Our results show that we are far from this ideal situation, since inter-practice variation could not be explained by the used demographic or case mix indicators in the present study. In addition, we hardly found any support for the idea that practice size or the presence of auxiliary staff in the practice would be an important factor. These results suggests that much can be improved in the detection of hypertension and diabetes mellitus in general practice in the Netherlands. In addition, it leaves us empty handed with respect to possible solutions; so far the variation seems to be random.

Of course this study has its limitations. We could not, for example, include all relevant risk factors for hypertension and diabetes mellitus. Smoking status, use of alcohol, body mass index, physical activity and family history of cardiovascular diseases and diabetes mellitus, are not recorded by GPs in a systematic way and therefore could not be used in this study. The same applies to a more detailed measurement of socio-economic status or deprivation and ethnic background. We could only use type of health care insurance as an indicator for socio-economic status, since this is the only socioeconomic parameter which is recorded in the electronic medical records. It is possible that our results would have been different if these risk factors were included. Furthermore, most of the symptoms and diseases used in the statistical models had a low prevalence, which makes discrimination between practices more difficult. Also at practice level (or preferably even a separate GP level), a number of possibly relevant factors could not be included. It would be interesting to investigate the effect of age, gender and years of experience of the GP. However, this was not possible since GPs in group practices and health center usually do not have personal patient lists. Moreover, it is possible that differences in the pro-active attitude of GPs in the studied disease could explain a part of the remaining variance. It would be interesting to compare our results with identical analyses of GP data in the UK to establish whether financial incentives from the Quality and Outcomes Framework (QOF) would reduce the inter-practice variation [18].

In spite of these limitations, however, we believe the most important and plausible factors to account for between practice variation have been included in this study. And yet a large amount of variation remains, suggesting that much can be improved in the detection of two of the major diseases of this time.

## Conclusion

It can be concluded that even after adjustment for patient and practice characteristics, there is a wide difference between general practices in the prevalence rates of diagnosed hypertension and diabetes mellitus. The reasons for this wide variation are unknown and further research in this area is needed.

## Competing interests

The authors declare that they have no competing interests.

## Authors' contributions

MMJN performed the study according to protocol, analyzed the data and wrote the manuscript. FGS and RAV initiated and supervised the study and developed the study protocol. All authors read and approved the final manuscript.

## Pre-publication history

The pre-publication history for this paper can be accessed here:



## Supplementary Material

Additional File 1 **Table 3 and 4.** Click here for file

## References

[B1] Zimmet P, Alberti KG, Shaw J (2001). Global and societal implications of the diabetes epidemic. Nature.

[B2] Kearney PM, Whelton M, Reynolds K, Muntner P, Whelton PK, He J (2005). Global burden of hypertension: analysis of worldwide data. Lancet.

[B3] Whelton PK (1994). Epidemiology of hypertension. Lancet.

[B4] Ritz E, Rychlik I, Locatelli F, Halimi S (1999). End-stage renal failure in type 2 diabetes: A medical catastrophe of worldwide dimensions. Am J Kidney Dis.

[B5] Walma EP, Thomas S, Prins A, Grundmeyer HGLM, Laan JR van der, Wiersma Tj (2003). NHG-Standaard Hypertensie (derde herziening). Huisarts Wet.

[B6] Rutten GEHM, Verhoeven S, Heine RJ, de Grauw WJC, Cromme PVM, Reenders K, van Ballegooie E, Wiersma Tj (1999). NHG-Standaard Diabetes Mellitus Type 2 (eerste herziening). Huisarts Wet.

[B7] Goyder E, Hammersley V (2003). Explaining variations in reported diabetes prevalence in general practice: how much variation is explained by differences between practice populations?. Br J Gen Pract.

[B8] Meadows P (1995). Variation of diabetes mellitus prevalence in general practice and its relation to deprivation. Diabet Med.

[B9] Whitford DL, Griffin SJ, Prevost AT (2003). Influences on the variation in prevalence of type 2 diabetes between general practices: practice, patient or socioeconomic factors?. Br J Gen Pract.

[B10] Khunti K, Goyder E, Baker R (1999). Collation and comparison of multi-practice audit data: prevalence and treatment of known diabetes mellitus. Br J Gen Pract.

[B11] Hooker RC, Cowap N, Newson R, Freeman GK (1999). Better by half: hypertension in the elderly and the 'rule of halves': a primary care audit of the clinical computer record as a springboard to improving care. Fam Pract.

[B12] Verheij RA, van Dijk CE, Abrahamse H, Davids R, Hoogen H van den, Braspenning J, van Althuis T (2008). Landelijk Informatienetwerk Huisartsenzorg Feiten en cijfers over huisartsenzorg in Nederland.

[B13] Verheij R, Zee J van der, Westert GP, Jabaaij L, Schellevis FG (2006). Collecting information in general practice: 'just by pressing a single button'?. Morbidity, performance and quality in primary care Dutch general practice on stage Oxon (UK).

[B14] Lamberts H, Wood M (1987). International Classification of Primary Care.

[B15] (2007). WHO Collaborating Centre for Drug Statistics Methodology. http://www.whocc.no/atcddd.

[B16] Snijders TAB, Bosker RJ (1999). Multilevel analysis An introduction to basic and advanced multilevel modeling.

[B17] Goldstein H, Rabash J, Plewis I, Draper D, Browne W, Yang M, Woodhouse G, Healy M (1998). A user's guide to MLwiN Multilevel Models Project.

[B18] Roland M (2004). Linking physicians' pay to the quality of care–a major experiment in the United kingdom. N Engl J Med.

