# Supplementary material for: Inter-practice variation in diagnosing hypertension and diabetes mellitus: a cross-sectional study in general practice
Source: BMC Fam Pract. 2009 Jan 21;10:6. doi: 10.1186/1471-2296-10-6 (PMC2632987; doi:10.1186/1471-2296-10-6)
Supplement: Additional File 1 — Table 3 and 4. [file 1471-2296-10-6-S1.doc]

Table 3: Inter-practice variation of hypertension

|  | **Model 1** | **Model 2** | | **Model 3** | |
| --- | --- | --- | --- | --- | --- |
|  | **B (standard error)** | **B (standard error)** | **OR (95%CI)** | **B (standard error)** | **OR (95%CI)** |
| ***Intercept*** | -1.816 (0.045) | -2.073 (0.041) | --- | -2.075 (0.040) | --- |
|  |  |  |  |  |  |
| ***Patient characteristics*** |  |  |  |  |  |
| Age (years) | --- | 0.091 (0.001) | **1.10 (1.09 -1.10)** | 0.091 (0.001) | **1.10 (1.09 - 1.10)** |
| Age^2^ (years^2^) | --- | -0.0013 (0.0001) | **1.00 (1.00 -1.00)** | -0.0013 (0.0001) | **1.00 (1.00 - 1.00)** |
| Age^3^ (years^3^) | --- | -0.000004 (0.000002) | **1.00 (1.00 - 1.00)** | -0.000004 (0.000002) | **1.00 (1.00 - 1.00)** |
| Gender (reference: Male) | --- | -0.300 (0.016) | **0.74 (0.72 - 0.76)** | -0.300 (0.016) | **0.74 (0.72 - 0.76)** |
| Type of health care insurance (reference: Public) | --- | -0.070 (0.017) | **0.93 (0.90 - 0.96)** | -0.070 (0.017) | **0.93 (0.90 - 0.96)** |
| Symptoms of cardiovascular disease (K01-K29) | --- | 0.461 (0.030) | **1.59 (1.50 - 1.68)** | 0.462 (0.030) | **1.59 (1.50 - 1.68)** |
| Ischemic heart disease with angina (K74) | --- | 0.545 (0.043) | **1.72 (1.59 - 1.87)** | 0.545 (0.043) | **1.73 (1.59 - 1.88)** |
| Heart failure (K77) | --- | 0.105 (0.052) | **1.11 (1.00 - 1.23)** | 0.105 (0.052) | **1.11 (1.00 - 1.23)** |
| Stroke / cerebro-vasculair accident (K90) | --- | 0.817 (0.063) | **2.26 (2.00 - 2.56)** | 0.818 (0.064) | **2.27 (2.00 - 2.57)** |
| Atherosclerosis / PVD (K92) | --- | 0.477 (0.081) | **1.61 (1.38 - 1.89)** | 0.478 (0.081) | **1.61 (1.38 - 1.89)** |
| Obesity (T82) | --- | 1.260 (0.097) | **3.53 (2.92 - 4.26)** | 1.262 (0.097) | **3.53 (2.92 - 4.27)** |
| Overweight (T83) | --- | 0.736 (0.121) | **2.09 (1.65 - 2.64)** | 0.737 (0.121) | **2.09 (1.65 - 2.65)** |
| Lipid disorder (T93) | --- | 1.108 (0.031) | **3.03 (2.85 - 3.22)** | 1.110 (0.031) | **3.03 (2.86 - 3.22)** |
| Urinary disease | --- | 0.381 (0.065) | **1.46 (1.29 - 1.66)** | 0.381 (0.065) | **1.46 (1.29 - 1.66)** |
| Diabetes mellitus | --- | 0.956 (0.024) | **2.60 (2.48 - 2.72)** | 0.958 (0.024) | **2.61 (2.49 - 2.73)** |
|  |  |  |  |  |  |
| ***Practice characteristics*** |  |  |  |  |  |
| Practice type (Reference: Single-handed) |  |  |  |  |  |
| Duo | --- | --- | --- | 0.042 (0.101) | 1.04 (0.86 - 1.27) |
| Group | --- | --- | --- | -0.069 (0.114) | 0.93 (0.75 - 1.17) |
| Health centre | --- | --- | --- | 0.064 (0.144) | 1.07 (0.80 - 1.41) |
| Presence of a nurse practitioner | --- | --- | --- | 0.016 (0.084) | 1.02 (0.86 - 1.20) |
|  |  |  |  |  |  |
| ***Model characteristics*** |  |  |  |  |  |
| Variance at practice level (standard error) | 0.112 (0.021) | 0.086 (0.017) | | 0.085 (0.017) | |
| Inter-practice variation (per 1000 persons) | 77.9 – 238.7 | 66.1 – 182.7 | | 66.3 – 181.7 | |
| ***OR’s in bold are statistical significant (p<0.05)*** | | | | | |

Table 4: Inter-practice variation of diabetes mellitus

|  | **Model 1** | **Model 2** | | **Model 3** | |
| --- | --- | --- | --- | --- | --- |
|  | **B (standard error)** | **B (standard error)** | **OR (95%CI)** | **B (standard error)** | **OR (95%CI)** |
| ***Intercept*** | -2.785 (0.048) | -3.215 (0.045) | --- | -3.220 (0.043) | --- |
|  |  |  |  |  |  |
| ***Patient characteristics*** |  |  |  |  |  |
| Age (years) | --- | 0.071 (0.001) | **1.07 (1.07 - 1.08)** | 0.071 (0.001) | **1.07 (1.07 - 1.08)** |
| Age^2^ (years^2^) | --- | -0.0007 (0.0001) | **1.00 (1.00 - 1.00)** | -0.0007 (0.0001) | **1.00 (1.00 - 1.00)** |
| Age^3^ (years^3^) | --- | -0.000007 (0.000002) | **1.00 (1.00 - 1.00)** | -0.000008 (0.000002) | **1.00 (1.00 - 1.00)** |
| Gender (reference: Male) | --- | 0.112 (0.022) | **1.12 (1.07 - 1.17)** | 0.112 (0.022) | **1.12 (1.07 - 1.17)** |
| Type of health care insurance (reference: Public) | --- | -0.395 (0.025) | **0.67 (0.64 - 0.71)** | -0.395 (0.025) | **0.67 (0.64 - 0.71)** |
| Ischemic heart disease with angina (K74) | --- | 0.199 (0.049) | **1.22 (1.11 - 1.34)** | 0.199 (0.050) | **1.22 (1.11 - 1.34)** |
| Heart failure (K77) | --- | 0.564 (0.056) | **1.76 (1.57 - 1.96)** | 0.564 (0.056) | **1.76 (1.57 - 1.96)** |
| Stroke / cerebro-vasculair accident (K90) | --- | 0.377 (0.070) | **1.46 (1.27 - 1.67)** | 0.377 (0.070) | **1.46 (1.27 - 1.67)** |
| Atherosclerosis / PVD (K92) | --- | 0.295 (0.094) | **1.34 (1.12 - 1.61)** | 0.295 (0.094) | **1.34 (1.12 - 1.61)** |
| Excessive thirst (T01) | --- | 0.502 (0.294) | 1.65 (0.93 - 2.94) | 0.501 (0.294) | 1.65 (0.93 - 2.94) |
| Obesity (T82) | --- | 0.887 (0.118) | **2.43 (1.93 - 3.06)** | 0.887 (0.118) | **2.43 (1.93 - 3.06)** |
| Overweight (T83) | --- | 0.739 (0.145) | **2.09 (1.57 - 2.78)** | 0.740 (0.146) | **2.10 (1.57 - 2.79)** |
| Lipid disorder (T93) | --- | 1.023 (0.035) | **2.78 (2.60 - 2.98)** | 1.023 (0.035) | **2.78 (2.60 - 2.98)** |
| Retinopathy (F83) |  | 2.138 (0.292) | **8.48 (4.78 - 15.02)** | 2.138 (0.292) | **8.49 (4.79 - 15.05)** |
| Urinary disease | --- | 0.388 (0.077) | **1.47 (1.27 - 1.71)** | 0.389 (0.077) | **1.48 (1.27 - 1.72)** |
| Hypertension | --- | 0.975 (0.023) | **2.65 (2.53 - 2.78)** | 0.976 (0.023) | **2.65 (2.54 - 2.78)** |
|  |  |  |  |  |  |
| ***Practice characteristics*** |  |  |  |  |  |
| Practice type (Reference: Single-handed) |  |  |  |  |  |
| Duo | --- | --- | --- | 0.008 (0.103) | 1.01 (0.82 - 1.23) |
| Group | --- | --- | --- | 0.240 (0.115) | **1.27 (1.01 - 1.59)** |
| Health centre | --- | --- | --- | 0.069 (0.146) | 1.07 (0.81 - 1.43) |
| Presence of a nurse practitioner | --- | --- | --- | 0.025 (0.086) | 1.03 (0.87 - 1.21) |
|  |  |  |  |  |  |
| ***Model characteristics*** |  |  |  |  |  |
| Variance at practice level (standard error) | 0.124 (0.025) | 0.093 (0.019) | | 0.084 (0.017) | |
| Inter-practice variation (per 1000 persons) | 30.0 – 109.7 | 21.6 – 68.1 | | 22.2 – 65.8 | |
| ***OR’s in bold are statistical significant (p<0.05)*** | | | | | |
